# Data on the cenozoic pyrometamorphic rocks of NE Brazil

**DOI:** 10.1016/j.dib.2019.103848

**Published:** 2019-05-22

**Authors:** Zorano Sérgio de Souza, Chao Wang, Zhen-Min Jin, Jian-Wei Li, Junlong Yang, Nilson Francisquini Botelho, Rúbia Ribeiro Viana, Larissa dos Santos, Peng-Lei Liu, Wei Li

**Affiliations:** a State Key Laboratory of Geological Processes and Mineral Resources, China University of Geosciences, 388, Lumo Road, Wuhan, 430074, PR China; b Pós-Graduação em Geodinâmica e Geofísica (PPGG, UFRN) and Departamento de Geologia, Universidade Federal do Rio Grande do Norte, Avenida Senador Salgado Filho, 3000, Campus Universitário, 59078-970 Natal/RN, Brazil; c Faculty of Earth Resources, China University of Geosciences, 430074 Wuhan, PR China; d Instituto de Geociências, Universidade de Brasília, Campus Universitário Darcy Ribeiro, 70910-900 Brasília/DF, Brazil; e Instituto de Ciência e Tecnologia - Campus JK, Rodovia MGT 367 - km 583, nº 5000, Alto da Jacuba CEP 39100-000, Diamantina, Brazil; f Pós-Graduação em Geodinâmica e Geofísica (PPGG, UFRN), Avenida Senador Salgado Filho, 3000, Campus Universitário, 59078-970 Natal, Brazil; g Key Laboratory of Submarine Geosciences, The Second Institute of Oceanography, State Oceanic Administration, Hangzhou 310012, China

## Abstract

The data presented in this article are related to the research paper entitled “Pyrometamorphic aureoles of Cretaceous sandstones and shales by Cenozoic basic intrusions, NE Brazil: Petrographic, textural, chemical and experimental approaches” Souza et al., 2018. Here, we report the complete data set for natural minerals and rocks as well as for experimental runs. These data include detailed oxide composition of minerals and glassy groundmass of the samples studied from electron microprobe and scanning electron microscopy analyzes. Rock samples and minerals are separated according to the protolith (sandstone, shale), pyrometamorphic rock (dark and light buchites, and silica-rich types), intrusion (basalt, diabase) that induced the pyrometamorphic events, and experimental results (microphenocrysts, glass).

**Table:** Specifications table

Subject area	*Geology.*
More specific subject area	*Geochemistry and Petrology.*
Type of data	*Tables, figures.*
How data was acquired	*Electron microprobe, Scanning electron microscope, experimental runs.*
Data format	*Analyzed data.*
Experimental factors	*Samples for the experimental runs were encapsulated in a graphite tube and then placed in a Pt capsule. A quarter of the capsule was cut along the cylindrical axis using a low-speed diamond saw. The remaining part was mounted in epoxy and polished to expose the sample.*
Experimental features	*The experiments were conducted in a 150-ton non-end-loaded piston-cylinder (Quickpress 3.0) apparatus. Experiments were pressurized to* 3 kbar *and at temperatures of 1200, 1100 and 1000°C for different runs.*
Data source location	*The region under investigation is situated* 70 km NW *Natal, NE Brazil. The center of the area has the WGS84 geographic coordinates 36.3 degrees West and 5.5 degrees South.*
Data accessibility	*The complete data set is with this article.*
Related research article	Souza, Z.S., Wang, C., Jin, Z.M., Li, J.W., Yang, J., Botelho, N·F., Viana, R.R., Santos, L., Liu, P.L., Li, W., 2018. Pyrometamorphic aureoles of Cretaceous sandstones and shales by Cenozoic basic intrusions, NE Brazil: Petrographic, textural, chemical and experimental approaches. Lithos 326–327, 90–109 [2].

**Table:** 

**Value of the data** • The chemistries of buchites and experimental runs for these kinds of rocks are not so easily found in the published literature. • The good agreement between the studied natural rocks (sedimentary protoliths transitioning to buchites along the contact with shallow basic intrusions) and the experimental runs permitted constraining both temperature and depth of intrusion of the thermal effect. • The complete data set amount to 397 spots analyzed. • They can be used for several purposes, e.g., statistical investigation of chemical variation of minerals and groundmass of the thermally affected rocks (dark and light buchites, silica-rich rocks) and constructing graphical plots other than the ones reported in [2] for comparison with results published on the literature.

## 1 Data

We present oxide composition data for the following groups of samples: (i) minerals of buchites (Table 1); (ii) minerals and glasses produced by experiments of melting of sandstone and shale under 3 kbar and temperatures of 1000–1200 °C followed by quenching (Table 2); (iii) minerals of basalts and diabase intrusions responsible for the pyrometamorphic event (Table 3); (iv) whole rock composition of the glassy groundmass of dark and light buchites, and silica-rich rocks (Table 4).

## 2 Experimental design, materials, and methods

### 2.1 Analytical methods and procedures

Analytical methods and procedures for mineral chemistries (electron microprobe analyzes, scanning electron microscopy) are described in [1].

### 2.2 Apparatus and methodology used for experimental fusion

**Fig. 1:**
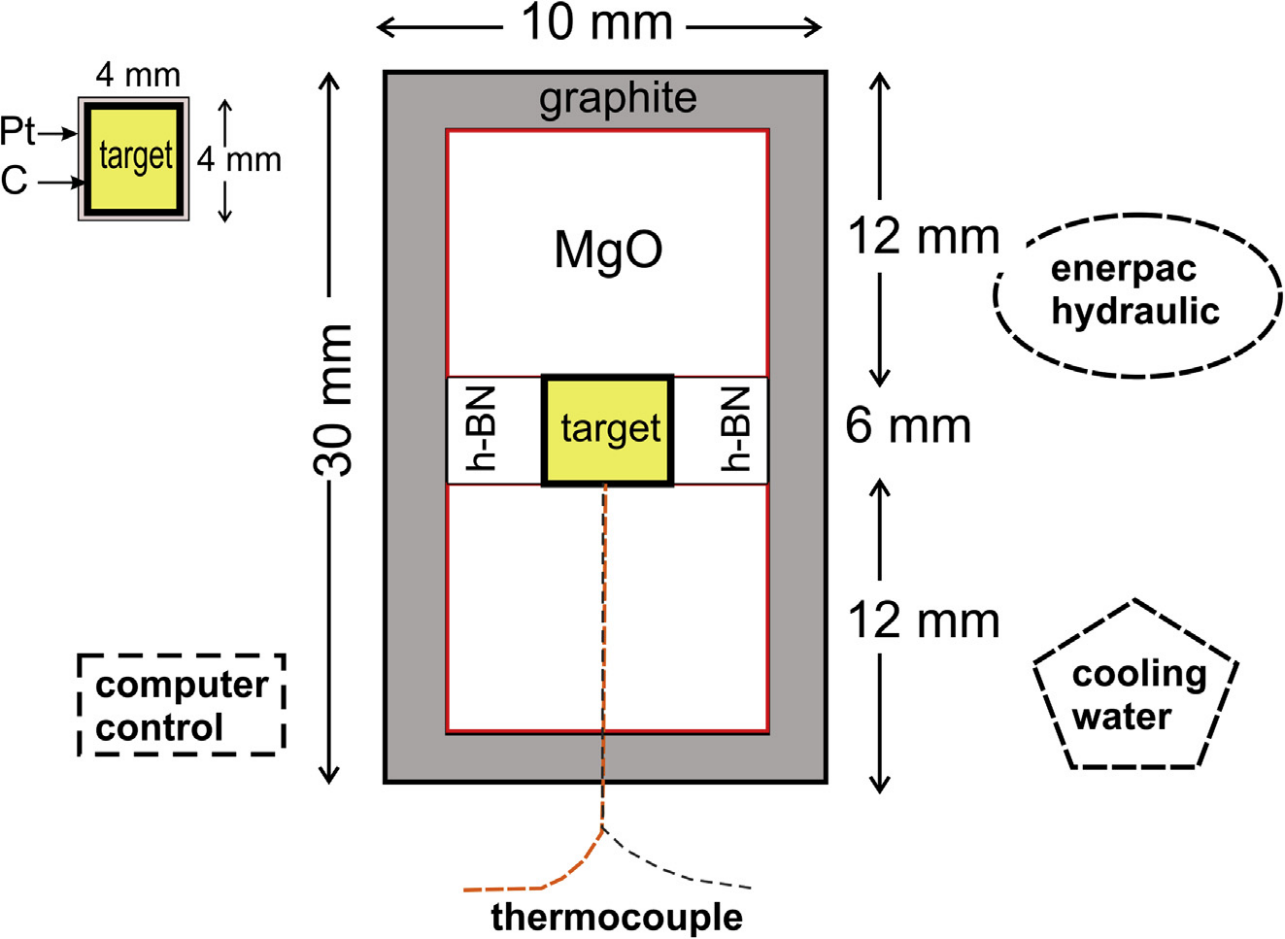
Geometric apparatus for the experiments. Modified and adapted after [1].

The experimental apparatus used is illustrated in Fig. 1. The experiments were conducted in a 150 ton non-end-loaded piston-cylinder (Quickpress 3.0) apparatus at the State Key Laboratory of Geological Processes and Mineral Resources of the China University of Geosciences, in Wuhan. The assembly consists of a Pt capsule sandwiched between two Boron Nitride (h-BN) rods in a graphite, Pyrex and salt sleeve. The Pt capsule was separated by a short h-BN sleeve from the graphite heater. The starting material (shale, sandstone, basalt, all with < ∼40 μm and ∼0.7 g total weight) was encapsulated in a graphite tube (2.2 mm internal diameter, 4.4 external diameter), which was then placed into a Pt capsule (4.5 mm internal diameter, 5.0 mm external diameter). The experimental temperature was monitored by inserting a W5Re-W26Re thermocouple into the high-pressure cell. The experiments were pressurized to 3 kbar and temperatures of 1200, 1100 and 1000 °C for different runs. They were ended by turning off the power to the press, resulting in quenching to below 200 °C within 10 s before the pressure was released. A quarter of the capsule was cut along the cylindrical axis using a low-speed diamond saw. The remaining part was mounted in epoxy and polished to expose the sample. After polishing and optical examination, identification of glass and fine-grained crystalline phases were done using a Quanta™ 450 FEG scanning electron microscope and points imaged by back-scattered electrons.
