# Supplementary material for: Data on the cenozoic pyrometamorphic rocks of NE Brazil
Source: Data Brief. 2019 May 22;25:103848. doi: 10.1016/j.dib.2019.103848 (PMC6603798; doi:10.1016/j.dib.2019.103848)
Supplement: Supplementary file 1 — Multimedia component 1 [file mmc1.pdf]

Conflict of Interest and Authorship Conformation Form

We confirm the following:

- All authors have participated in (a) conception and design, or analysis and interpretation of the data; (b) drafting the article or revising it critically for important intellectual content; and (c) approval of the final version.

- This manuscript has not been submitted to, nor is under review at, another journal or other publishing venue.

- The authors have no affiliation with any organization with a direct or indirect financial interest in the subject matter discussed in the manuscript.

- No authors have affiliations with organizations with direct or indirect financial interest in the subject matter discussed in the manuscript.

Sincerely yours

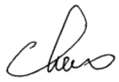

Chao Wang
